# Evaluation of prediction models for the staging of prostate cancer

**DOI:** 10.1186/1472-6947-13-126

**Published:** 2013-11-15

**Authors:** Susie Boyce, Yue Fan, Ronald William Watson, Thomas Brendan Murphy

**Affiliations:** 1 UCD School of Medicine and Medical Science, University College Dublin, Dublin, Ireland; 2 UCD School of Mathematical Sciences, University College Dublin, Dublin, Ireland; 3 UCD School of Biomolecular and Biomedical Science, University College Dublin, Dublin, Ireland; 4 Conway Institute, University College Dublin, Belfield, Dublin 4, Ireland

**Keywords:** Prediction models, Model evaluation, Discrimination, Calibration, Prostate cancer

## Abstract

**Background:**

There are dilemmas associated with the diagnosis and prognosis of prostate cancer which has lead to over diagnosis and over treatment. Prediction tools have been developed to assist the treatment of the disease.

**Methods:**

A retrospective review was performed of the Irish Prostate Cancer Research Consortium database and 603 patients were used in the study. Statistical models based on routinely used clinical variables were built using logistic regression, random forests and k nearest neighbours to predict prostate cancer stage. The predictive ability of the models was examined using discrimination metrics, calibration curves and clinical relevance, explored using decision curve analysis. The N = 603 patients were then applied to the 2007 Partin table to compare the predictions from the current gold standard in staging prediction to the models developed in this study.

**Results:**

30% of the study cohort had non organ-confined disease. The model built using logistic regression illustrated the highest discrimination metrics (AUC = 0.622, Sens = 0.647, Spec = 0.601), best calibration and the most clinical relevance based on decision curve analysis. This model also achieved higher discrimination than the 2007 Partin table (ECE AUC = 0.572 & 0.509 for T1c and T2a respectively). However, even the best statistical model does not accurately predict prostate cancer stage.

**Conclusions:**

This study has illustrated the inability of the current clinical variables and the 2007 Partin table to accurately predict prostate cancer stage. New biomarker features are urgently required to address the problem clinician’s face in identifying the most appropriate treatment for their patients. This paper also demonstrated a concise methodological approach to evaluate novel features or prediction models.

## Background

Prostate cancer (PCa) is the most common cancer in European and North American men, and the second most common cause of male cancer deaths [1]. There are dilemmas associated with the diagnosis and prognosis of PCa which has lead to the over diagnosis and over treatment of the disease [2]. However, new treatments such as active surveillance are being introduced to overcome these issues [3-6].

Prediction tools for PCa have been developed to assist in the accurate diagnosis and treatment of the disease, and address a wide variety outcomes; e.g. the Partin tables [7-10], Partin nomogram [11], Kattan and Stephenson nomograms [12-14], D’Amico risk classification [15], CAPRA score [16] and many others [17-19]. For the prediction of stage at radical prostatectomy (RP), the Partin tables not only represent the most common prediction tool used by clinicians, but have also undergone extensive validation in a number of cohorts [20-26]. The Partin table uses clinical stage based on digital rectal exam (DRE), Gleason score (GS) of the prostate needle biopsy [27-31], and serum prostate specific antigen (PSA) to predict stage at RP. PCa stage indicates the extent or location of the cancer, and can be categorized as; organ confined (OC), extracapsular extension (ECE), seminal vesicle invasion (SVI) and/or lymph node involvement (LNI). Non-organ confined (NOC) disease represents any stage which extends beyond the prostate organ, i.e. ECE, SVI or LNI.

While the Partin table is well used by clinicians, excluding this, few other prediction tools are used in a clinical setting. To overcome this issue, external validation of prediction models are ongoing. External validations which validate and compare two or more models are particularly useful. Chun et al. used this approach and compared five logistic regression (LR) based nomograms with other LR based models, namely look up table, classification and regression tree, artificial neural networks and risk group stratification [32]. However, each set of models being compared was developed in different patient cohorts and different outcomes were compared, i.e. nomogram for BCR and classification and regression tree for BCR, nomogram for stage and look up table for stage.

The Partin table was developed using multivariate logistic regression (MLR), however it isn’t known whether other statistical modelling techniques would have been more accurate to use with this type data. By extending the work of Chun et al. and Partin et al., the aim of this study is to explore a number of classification techniques rather than just LR, each predicting the same outcome and developed and tested in one cohort of patients, using the same variables as those used in the Partin tables. We also aim to explore methods to evaluate prediction models, such as discrimination and calibration metrics, as well as decision curve analysis.

## Methods

### Study population

A retrospective review was performed of the Irish Prostate Cancer Research Consortium (PCRC) database. The PCRC was founded in 2003, and is a multi-disciplinary trans-institutional collaboration. Patient samples were sourced from four institutions; three tertiary referral centres and one private hospital. Eight consultant urologists and four distinct pathology departments are involved in the acquisition and grading of prostatic tissue. Ethical approval was awarded in each hospital (Mater Misericordiae University Hospital, St James’s Hospital, Beaumont Hospital, Mater Private Hospital). Written informed consent was obtained from study participants. Inclusion criteria for this study were availability of pre-operative serum PSA, trans-rectal ultrasound guided needle biopsy Gleason Score [27-31], clinical T stage using TNM staging [33] identified by DRE and the corresponding RP pathology reports. All study participants had pathologically confirmed prostatic adenocarcinoma. Between February 2002 and October 2011, data relating to 705 patients who underwent RP was collected through the PCRC. A total of 102 patients were excluded due to benign prostatic hyperplasia (BPH) and missing data. This left a total of 603 patients.

### Clinical and pathological assessment

The clinical stage was stratified as T1c (DRE negative) or T2 (DRE positive) [33]. Recording of the sub-stratification of T2 was not available for the analysis. The Gleason scoring system was used for needle biopsy grading [27-31]. RP specimens were assigned as organ confined (OC) if the tumour can be felt on examination, but has not spread outside the prostate, extra capsular extension (ECE) if the tumour has spread through the prostatic capsule on one or both sides, seminal vesicle invasion (SVI) if the tumour has invaded one or both seminal vesicles and lymph node involvement (LNI) if the pelvic lymph nodes exhibited prostate cancer [33]. Patients were then re-stratified as organ confined (OC) or non-organ confined (NOC), where NOC represents any pathological stage which is not OC.

### Statistical analysis

Patient information included pre-operative PSA, clinical stage based on DRE, biopsy Gleason score (GS), age and family history. Descriptive statistics focused on frequencies and proportions for categorical variables. Means, medians, and ranges were reported for continuous data and error measures were reported as 95% confidence intervals (CI). The parametric independent samples *t*-test and non-parametric Mann Whitney U test were used to examine the statistical significance of differences in means for normal and non-normal data respectively. Chi-square test was used to compare frequencies for categorical data.

Seven statistical and algorithmic classification techniques were used to develop models to predict stage at RP. These included logistic regression, linear discriminant analysis, classification and regression trees, k nearest neighbours, artificial neural networks, support vector machines and random forests. The objective of a classification model is to classify patients in two or more groups based on a predicted outcome associated with each patient. On examination of the individual model fit for each of the seven classification techniques, three models were chosen for further analysis and model evaluation: logistic regression [34], random forests [35] and k nearest neighbours [36].

**Figure 1:**
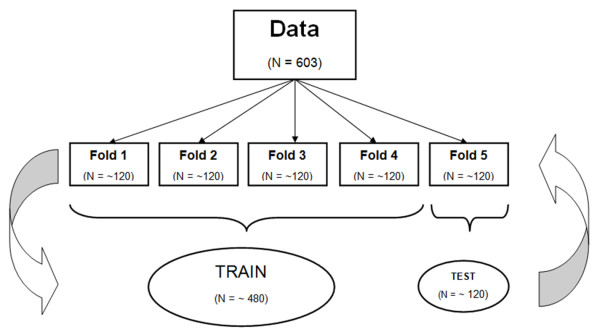
**Illustration of 5-fold cross validation.** The dataset is randomly split into five subgroups (called folds). Four folds are merged together to form a training dataset. The 5^th^ fold is used as the testing dataset. This process is then repeated five times.

The data was prepared for modelling using 5-fold cross validation (Figure 1). 5-fold cross validation involves randomly dividing the data into five evenly sized subgroups. Each group is called a fold. A model is then constructed using the data from the first four folds and applied to the fifth group. The model building and validation process is repeated five times with each fold of patients used once as the validation set. This results in no patient being used to both develop and test the model [37].

Model evaluation was carried out by examining calibration, discrimination and decision curve analysis [37-41]. The calibration of the models was measured using calibration curves [39]. Calibration measures how close the predicted probabilities are to actual probabilities. A calibration curve plots predictions on the x-axis and the true outcome on the y-axis. Due to the fact that the actual outcomes are 0 and 1, Loess smoothing [42,43] was used to estimate the observed probabilities of the outcome in relation to the predicted probabilities. The discriminate ability of the models were compared by formulation of sensitivity [44], specificity [44], positive predictive value (PPV) [45], negative predictive value (NPV) [45], Youden index [46,47], Brier score [48] and area under the curve (AUC) values [49,50]. The discriminate ability of a model measures how well the model discriminates between patients with and without the outcome. The AUC value provides us with a probability that the model will correctly identify which of two individuals with different outcomes actually has the disease.

However, there has been much criticism of the AUC value in the last number of years [51,52]. This is due to the fact that patient’s do not present to a clinician’s office in pairs, one of whom has NOC disease and the other with OC disease. There is also concern regarding what the necessary AUC value should be for a model to be considered ‘clinically useful’. To overcome these issues, decision curve analysis was used to measure the clinical relevance of the three models [37,40,41]. Decision curve analysis is a method for evaluating and comparing prediction models that incorporates clinical consequences. It is based on the principle that the probability at which a physician would advise treatment is informative on how the physician and patient weigh the harms of false-positive results in comparison with the harms of false-negative results. This probability is referred to as the threshold probability (P_t_). This threshold probability (P_t_) can then be used to derive the net benefit of the model across different threshold probabilities, where:

(1) $$\mathrm{Net}\;\mathrm{Benefit}=\frac{\mathrm{True}\;\mathrm{Positive}\;\mathrm{Count}}{n}-\frac{\mathrm{False}\;\mathrm{Positive}\;\mathrm{Count}}{n}\cdot \left(\frac{P_{t}}{1-P_{t}}\right)$$

Plotting net benefit against threshold probability results the ‘decision curve’. The decision curve gives the expected net benefit per patient relative to assuming all patients have OC disease, the expected benefit associated with assuming all patients have NOC and the expected benefit associated with using the classification model. The interpretation of net benefit is the model with the highest net benefit should be chosen.

The patient’s clinical data was also applied to the 2007 Partin table for ECE [7] in order to evaluate how well this prediction tool can predict stage at RP compared to the three classification models developed in this study. The predictions from the Partin tables were measured for discrimination.

Statistical analysis was performed using R software, version 2.14.0 with the following packages: ‘car’, ‘boot’, ‘rpart’, ‘randomForest’, ‘class’, ‘e1071’, ‘MASS’, ‘nnet’, ‘ROCR’, ‘pROC’, ‘Hmisc’, ‘rms’, ‘gmodels’, ‘gplots’, ‘epicalc’.

## Results

The clinical and pathological characteristics of the N = 603 PCRC patient cohort are given in Table 1. Average patient age was 61 (C.I: 60.4, 61.6) years (median 62, range 42–74). Average PSA value was 7.96 (C.I: 7.57, 8.26) ng/ml (median 7.0, range 0.7-40). Most patients had clinical stage T1c (44.3%), biopsy Gleason score 6 (37.1%) and prostatectomy Gleason score 3 + 4 (39.3%). 54.9% had no family history of cancer, 20.1% had a history of cancer (excluding PCa) in the family and 25.0% had a family history of PCa. Most patients, 70%, had OC disease while the remaining 30% had NOC disease (Table 1). Of the NOC patients, 19% had ECE, 8% SVI and 3% LNI. Patients with NOC disease had a higher average PSA than OC patients (Mean: 8.6 ng/ml vs. 7.6 ng/ml), higher biopsy Gleason score (GS8: 9.9% vs. 4.6%), higher prostatectomy Gleason score (GS8: 12.5% vs. 4.7%) and were older (Mean: 62.2 years vs. 60.7 years). These findings were all statistically significant at the *P <* 0.05 level. No significant differences were recorded according to stage at RP for clinical stage or family history (both *P* > 0.05).

**Table 1:** Prostate cancer research consortium patient cohort characteristics

	**PCRC data**	**OC**	**NOC**	***p***
	**(n = 603)**	**(n = 427)**	**(n = 176)**	
Age (y)				
Mean (Median)	61.1 (62)	60.7 (61)	62.2 (63)	*0.01*
Range	42-74	42-74	42-74	
Family history (%)				
PCa history	151 (25.0)	107 (25.1)	46 (28.9)	*0.70*
Ca history	121 (20.1)	85 (19.9)	26 (16.4)	
No history	331 (54.9)	235 (55.0)	87 (54.7)	
Clinical stage, DRE (%)				
T1c	267 (44.3)	188 (44.1)	79(44.9)	*0.69*
T2a	144 (23.9)	99 (23.1)	45 (25.6)	
Not reported	192(31.8)	140 (32.8)	52 (39.5)	
PSA (ng/ml)				
Mean (Median)	7.96 (7.0)	7.6 (6.7)	8.6 (7.5)	*0.02*
Range	0.7 – 40	0.7 – 40	2.1 – 36	
Biopsy GS (%)				
5	96 (17.4)	77 (19.7)	19 (11.8)	*P < 0.001*
6	205 (37.1)	163 (41.7)	42 (26.1)	
3 + 4 = 7	153 (27.7)	103 (26.3)	50 (31.1)	
4 + 3 = 7	46 (8.3)	22 (5.7)	24 (14.9)	
8	34 (6.2)	18 (4.6)	16 (9.9)	
9	18 (3.3)	8 (2.0)	10 (6.2)	
Prostatectomy GS (%)				
5	59 (9.8)	46 (10.8)	13 (7.4)	*P < 0.001*
6	151 (25.0)	125 (29.2)	26 (14.8)	
3 + 4 = 7	237 (39.3)	181 (42.4)	56 (31.8)	
4 + 3 = 7	90 (14.9)	44 (10.3)	46 (26.1)	
8	42 (7.0)	20 (4.7)	22 (12.5)	
9	24 (4.0)	11 (2.6)	13 (7.4)	
Pathological stage				
OC	427 (70)	427 (70)	-	
ECE	111 (19)	-	111 (63)	
SVI	47 (8)	-	47 (27)	
LNI	18 (3)	-	18 (10)	

The Gleason score based on TRUS biopsy and the Gleason score recorded by pathology at RP were compared to measure the percentage of Gleason score upgrading or downgrading (Table 2). 52.9% of patients experience no Gleason upgrading or downgrading, i.e. the results of their TRUS biopsy were accurate. However, of the remaining 47.1% of patients, 34.1% experienced upgrading and 13.0% experienced down grading of their Gleason score. This indicates a 47% grading error based on TRUS biopsy.

**Table 2:** Percentage of Gleason score upgrading or downgrading

	**Biopsy GS**					
**N (%)**	**≤6**	**7 (3 + 4)**	**7 (4 + 3)**	**8**	**9-10**	**Total**
N	301	153	46	34	18	552
Decrease in GS	0	23 (15.1)	15 (32.6)	25 (73.5)	9 (50.0)	72 (13.0)
No change	170 (56.5)	92 (60.1)	16 (34.8)	5 (14.7)	9 (50.0)	292 (52.9)
Increase in GS	131 (43.5)	38 (24.8)	15 (32.6)	4 (11.8)	0	188 (34.1)

Seven prediction models were developed using logistic regression, linear discriminant analysis, classification and regression trees, k nearest neighbours, artificial neural networks, support vector machines and random forests. On examination of the individual model fit for each classification technique, the linear discriminant analysis, classification and regression trees, artificial neural networks and support vector machines models were excluded as these classification techniques were deemed inferior in this study. This resulted in three prediction models; a model developed using logistic regression, a model developed using random forests and a model developed using k nearest neighbours. Each of these models contains the same predictor variables (PSA, clinical stage and biopsy GS) and have all been developed using the same 5-fold cross validation approach.

The discriminate ability of the three models was measured using discrimination metrics including sensitivity, specificity, Youden index, positive predictive value (PPV), negative predictive value (NPV), Brier score and AUC values (Table 3). The logistic regression (LR) model illustrates a sensitivity of 0.647 and a specificity of 0.601, indicating that this model correctly identified 64.7% of patients who had NOC disease and 60.1% of patients who had OC disease, i.e. the model discriminates between both NOC patients and OC patients to the same ability. However, these values for sensitivity and specificity, although high relative to the other results in Table 3, are low based on the fact that a perfect model would achieve a sensitivity and specificity of 1. The Youden index for the LR model is calculated as a summation of the sensitivity and specificity minus 1; therefore due to the fact that both the sensitivity and specificity are reasonably good, the Youden index for this model (0.248) is reasonably good relative to the others in Table 3. The Youden index is a useful metric when there is no preference between sensitivity and specificity. The LR model had a PPV of 0.495 and NPV of 0.800, indicating that 49.5% of patients in the sample who were predicted as being NOC by the model actually had NOC disease and 80% of patients who were predicted as being OC actually had OC disease. It should be noted that, unlike sensitivity and specificity, NPV and PPV are affected by the prevalence of disease in the sample. In this study, the prevalence of having NOC disease is 30% and of having OC disease is 70% (Table 1). When the prevalence is low the PPV will be low, regardless of the sensitivity and specificity. The Brier score for the LR model is 0.173. The maximum Brier score for a model with a prevalence of 30% is approximately 0.21. A model with a Brier score of 0.21 indicates that there are large differences between the predicted probabilities and the actual outcome. The AUC value for the LR model is 0.622, which is reasonably good, but an AUC of 0.70 and above would be the minimum required to consider a model useful for clinical application. When comparing the LR model AUC with those from the other classification models and clinical variables in isolation (Table 3), the AUC of 0.622 for the LR model is the highest. This is closely followed by biopsy Gleason score (AUC = 0.618, Sens = 0.623, Spec = 0.613, PPV = 0.396, NPV = 0.799, Brier = 0.179). These results would indicate that biopsy Gleason score is by far the individual predictor variable with the highest discriminate ability. The integration of biopsy Gleason score with the other clinical variables into a LR model improves the ability to predict PCa stage at RP, but this improvement is minimal, highlighting the strength of biopsy Gleason score alone. Neither the random forests (RF) model (AUC = 0.605, Sens = 0.673, Spec = 0.457, PPV = 0.339, NPV = 0.771, Brier = 0.206) nor the K nearest neighbours (kNN) model (AUC = 0.570, Sens = 0.673, Spec = 0.457, PPV = 0.339, NPV = 0.771, Brier = 0.215) achieve better overall discrimination than biopsy Gleason score alone or the LR model.

**Table 3:** Discrimination of prediction models and individual clinical variables

	**Logistic regression**	**Random forests**	**K nearest neighbours**	**Biopsy Gleason score**	**PSA**	**Clinical stage**
**Sens**	0.647	0.477	0.673	0.623	0.678	0
**Spec**	0.601	0.714	0.457	0.613	0.446	1
**Youden**	0.234	0.192	0.129	0.235	0.124	0
**PPV**	0.495	0.410	0.339	0.396	0.340	NA
**NPV**	0.800	0.767	0.771	0.799	0.767	NA
**Brier**	0.173	0.206	0.215	0.179	0.195	0.208
**AUC**	0.622	0.605	0.570	0.618	0.571	0.572

**Figure 2:**
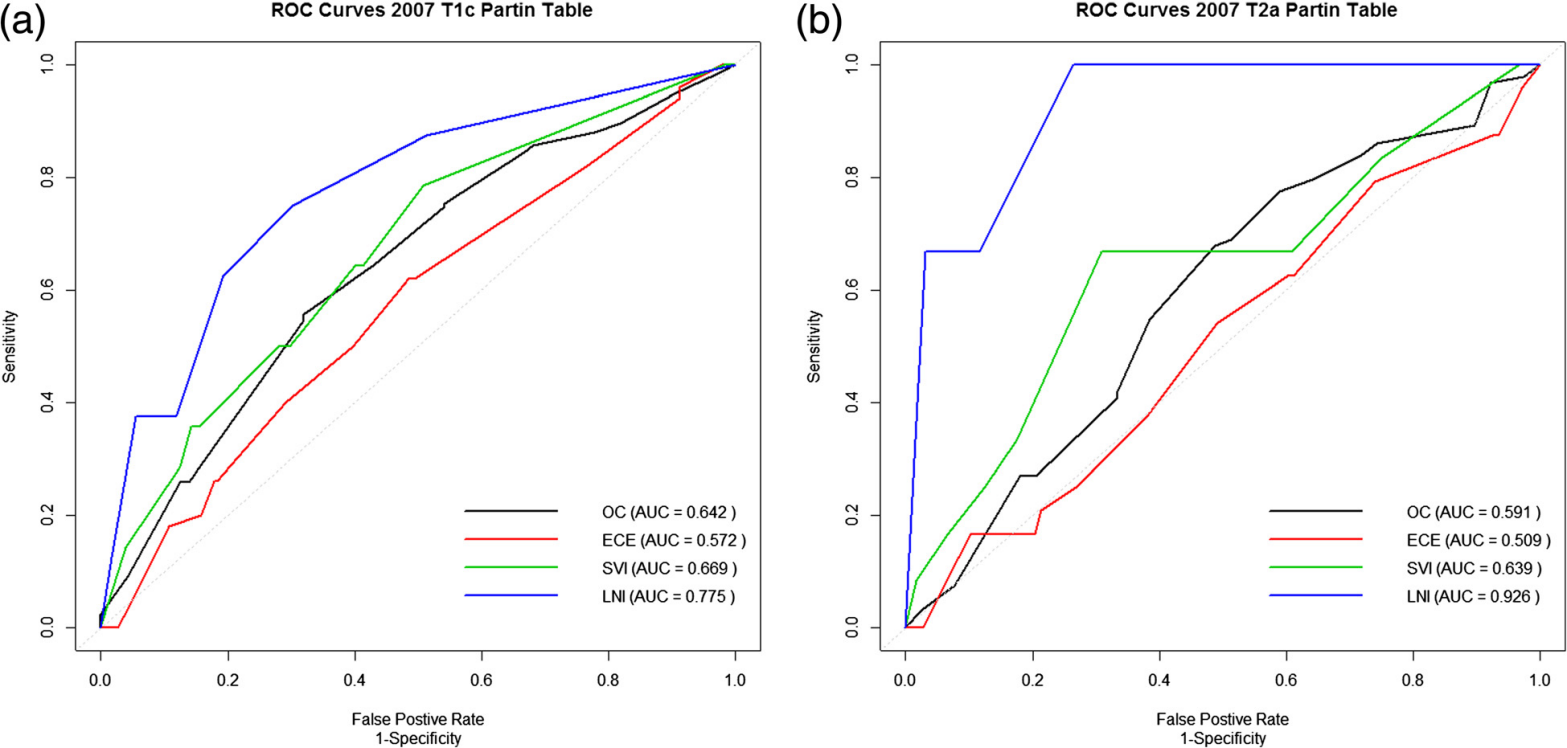
**Predictions from the 2007 Partin tables.** The patient’s data was applied to the 2007 **(a)** T1c and **(b)** T2a Partin tables and the discriminate ability measured using ROC curves and AUC values. The AUC values of the ECE predictions are lower than those of the logistic regression model developed in this study.

The discrimination of the 2007 Partin table was also measured (Figure 2). It should be noted that the Partin tables predict four stages at RP (OC, ECE, SVI and LNI), whereas the models developed in this study predict NOC disease, where NOC is made up of ECE, SVI and LNI. The majority of the NOC patients are made up of ECE, therefore the most appropriate Partin table prediction to look at in comparison to this study is the ECE predictions. The 2007 Partin table can predict ECE with an AUC value of 0.572 for patients with clinical stage T1c (Figure 2a) and 0.509 for patients with clinical stage T2a (Figure 2b).

**Figure 3:**
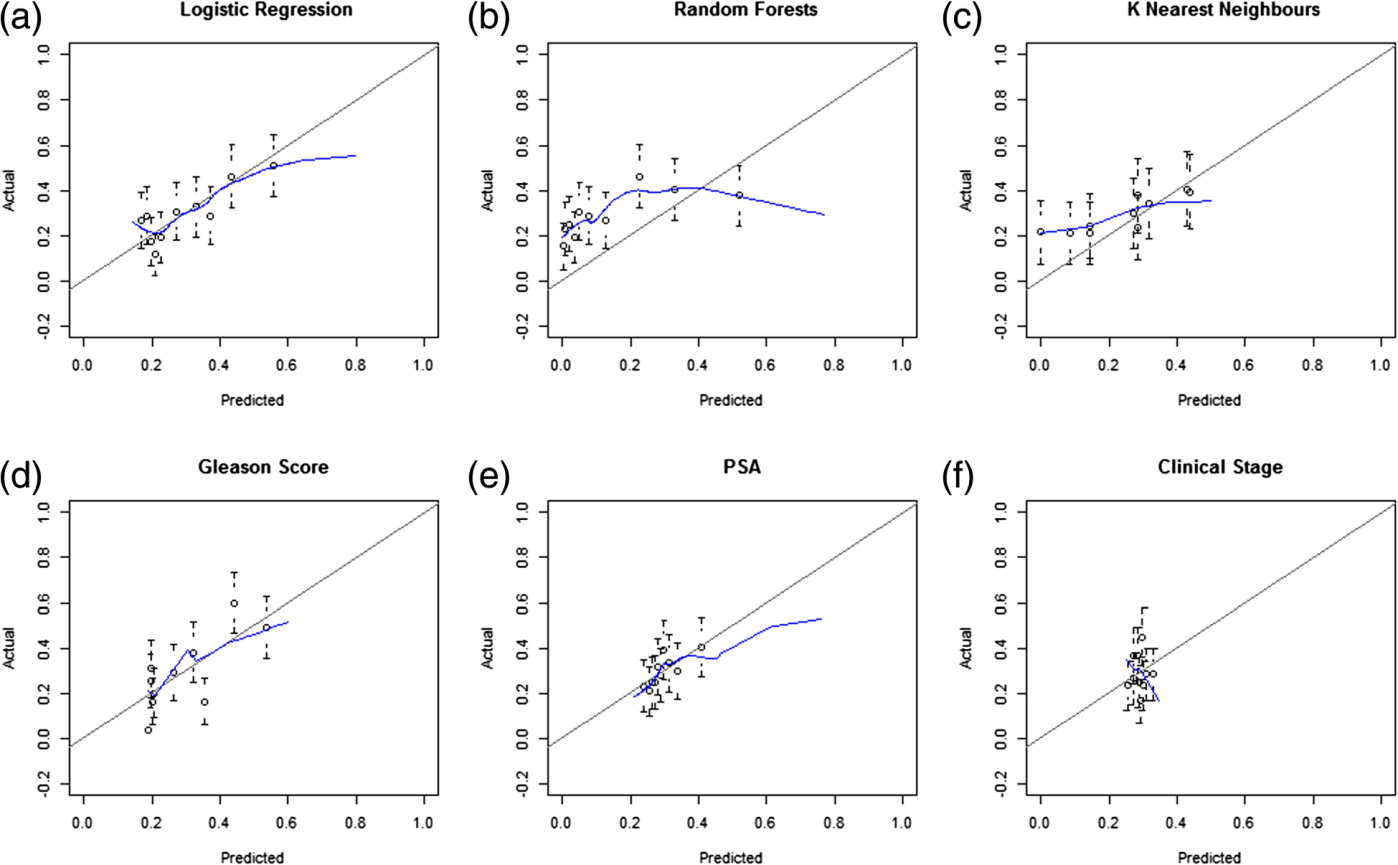
**Calibration curves which illustrate predicted probabilities on the x-axis and the actual outcome on the y-axis.** In this case the outcome is a binary variable hence Loess smoothing (blue line) was used to estimate the actual outcomes for **(a)** logistic regression model, **(b)** random forests model, **(c)** k nearest neighbours model, **(d)** biopsy Gleason score, **(e)** PSA and **(f)** clinical stage.

The calibration of each model was graphically measured by formulation of calibration curves (Figure 3a-f). The blue line represents the fit based on Loess smoothing. A model is well calibrated if the predicted probabilities or Loess smoothing fit (blue line) lie along the 45° line. Deviations away from this indicate mis–calibration. The error bars represent the 95% confidence interval for the predicted probabilities. The LR model (Figure 3a) is well-calibrated, although there appears to be very slight deviations from the 45° line at the very low and very high predicted probabilities, indicating that some of the lower predicted probabilities may slightly under estimate the true outcome and some of the higher predicted probabilities may slightly over-predict the true probability of the patient, but it should be noted that these deviations are minimal. The RF and kNN models illustrate some mis-calibration (Figure 3b-3c), indicating that the predicted probabilities for these models deviate from the true patient probability. Biopsy Gleason score (Figure 3d) is reasonably well-calibrated; although some of the error bars at predicted probabilities of approx 0.4 and 0.5 do not conform to Loess smoothing. PSA is reasonably calibrated (Figure 3e) although some clear over-prediction is occurring at higher probabilities based on Loess smoothing. The error bars indicate that the actua probabilities are well calibrated. The calibration curve for clinical stage (Figure 3f) illustrates how narrow the band of predicted probabilities is for the model built based on clinical stage (DRE) alone. The predicted probabilities vary between approx 0.25 and 0.35. Based on this it is difficult to examine the shape of the calibration of the error bars, although the Loess smoothing blue line clearly appears to be mis-calibrated but this may in fact be due to the narrowness of the range of predicted probabilities. Regardless of the fact that the predicted probability calibration based on the error bars looks reasonably good, the narrow range of the predicted probabilities indicates the weakness of the clinical stage model and this has also been shown in previous results (Table 3).

**Figure 4:**
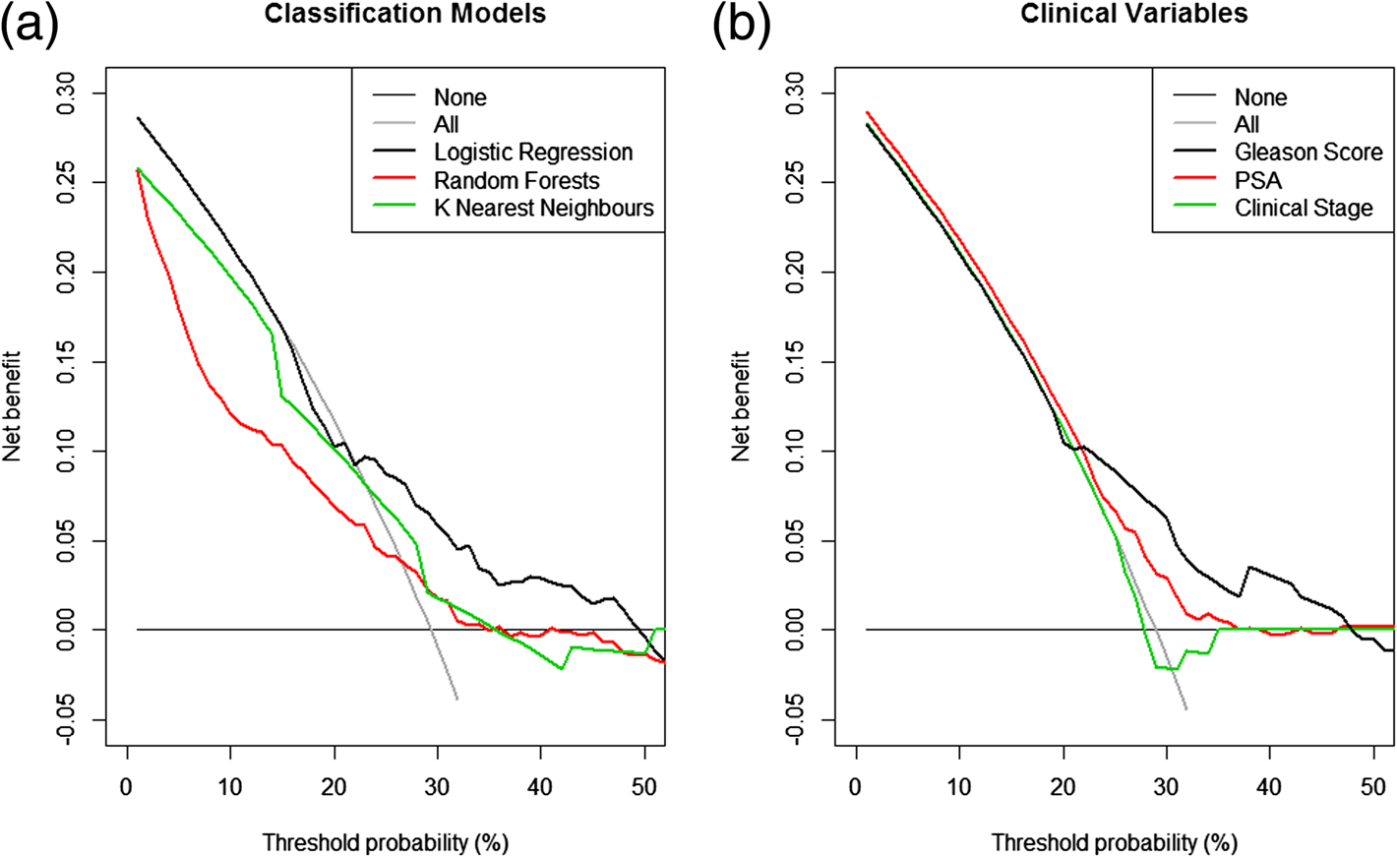
**Decision curves for (a) logistic regression model, random forests model and k nearest neighbours model and (b) biopsy Gleason score, PSA and clinical stage.** In each decision curve the solid, thin black line represents assuming no one has NOC PCa and the thin grey line represents assuming everyone has NOC PCa.

The results of decision curve analysis are compared by means of decision curves (Figure 4), with separate decision curves for the classification models (Figure 4a) and the independent clinical variables in isolation (Figure 4b). For both figures, the straight black line at y = 0 represents the decision curve for the strategy of treating no patients for NOC disease and the grey line represents the decision curve for the strategy of treating all patients for NOC disease. The LR model is superior to the RF and kNN models as it has the highest net benefit at the majority of threshold probabilities along the x-axis (Figure 4a). From the same figure, it is also clear that the LR model is well calibrated: for the majority of threshold probabilities, the model never does worse than treating everyone (grey line) and treating no one (thin black line at net benefit = 0), unlike the other two models (RF and kNN), again illustrating that LR is the superior model in terms of discrimination, calibration and now also clinical relevance. An advantage of decision curves is the ability to identify the range of probabilities at which a model will be clinically relevant. For example, a clinician could input a new patient’s clinical information into the model based on LR and calculate their predicted probability. The clinician would then refer to the decision curve and find the predicted probability along the x-axis and identify which prediction model has the highest net benefit at that point. If the LR model does not have the highest net benefit at that point, the LR model is not the most appropriate to use for this patient and an alternative (the model with the highest net benefit at that point) should be used instead. The RF and kNN models show mis-calibration at threshold probabilities between 0-25%. The range of threshold probabilities that these two models would be useful at is between 25-30%, however, at these threshold probabilities, the LR model would be the optimal prediction tool to use. The range of threshold probabilities that the LR model would be useful at is between 0-50%, and although there is a slight dip at approx 20%, at the majority of threshold probabilities this model has the highest net benefit. Of the individual clinical variables (Figure 4b), the Gleason score model appears to be the optimal model at threshold probabilities of 25% and above, below which the PSA model appears to be have a slightly higher net benefit. All of the models based on clinical variables in isolation appear to be reasonably well calibrated (except for clinical stage) as they are never worse than treating everyone and treating no one. Clinical stage (thick blue line) shows clear mis-calibration due to the fact that at threshold probabilities between approx 26-31%, the model is worse than treating everyone and this model also appears to be poorly discriminative due to the fact that between threshold probabilities of 30-35%, the model is worse than treating no one.

## Discussion

70% of patients had OC disease while the remaining 30% had NOC disease (Table 1). This represents a 30% staging error, as the entire study cohort were assumed to have OC disease and hence underwent RP. 47.1% of patient’s biopsy Gleason score was an incorrect estimate of their true Gleason score at RP, indicating that only 52.9% of patients did not experience an upgrading or downgrading of their Gleason score. Our group had previously shown a 42% Gleason score error between biopsy and RP in a smaller sample (N = 206) of the same patient cohort [24]. This level of upgrading or downgrading (42%) has been illustrated in other studies [53]. It was difficult to ascertain published figures for Gleason score upgrading or downgrading for the last number of years, particularly studies with a reasonably large sample size such as this one hence the result that 47.1% of patients experienced upgrading or downgrading of their biopsy Gleason score at RP is a significant finding of the paper.

The inclusion of the three clinical predictor variables into a statistical classification model provided a minimal improvement in predictive ability (discrimination, calibration and clinical relevance) compared to the model based on Gleason score in isolation, however, it was an improvement none-the-less. It is obvious that the statistical classification model is a welcome addition to PCa prediction, even more so due to the fact that the future of PCa staging is bound to contain complex new tests, biomarkers or features. There is no alternative to integrating multiple variables in a single prediction model [52]. This study has illustrated LR as a superior modelling technique.

Using the current clinical variables alone, excellent or even good discrimination, calibration and clinical utility will never be observed. Gleason score, PSA and clinical stage based on DRE do not contain enough information to accurately predict PCa stage at RP. New predictive features are urgently required for the prediction of PCa staging. The future of PCa prediction will likely involve the integration of novel biomarkers with existing clinical features. There are many ongoing biomarker discovery and validation studies, both published and in progress [54-63]. The modelling of such integrated data sets does not present a problem. This study has illustrated LR is as good and if not better than some of the newer more complex classification techniques. This is due in part to the fact that there are no complex relationships between PCa variables which need to be allowed for in a statistical model. The area which will require further, ongoing research is around methods to evaluate a new predictive marker/model. An initial framework to address this has been implemented in this study, an approach which examined discrimination, calibration and clinical relevance, based on previous work by Steyerberg et al. [39,64,65].

## Conclusion

This study has illustrated the inability of the current clinical variables to accurately predict PCa stage. This is in part due to the fact that the most predictive clinical variable, Gleason score, over or underestimates the true Gleason score at RP in 47.1% of patients. New biomarkers or features are urgently required to address the problem clinician’s face regarding accurately prognosticating the appropriate treatment for PCa patients. This paper has illustrated an approach which may be useful in the evaluation of such novel biomarkers or features, or prediction models in general.

## Abbreviations

PCa: Prostate cancer; RP: Radical prostatectomy; PSA: Prostate specific antigen; DRE: Digital rectal exam; TRUS: Transrectal ultrasound; OC: Organ confined; ECE: Extracapsular extension; SVI: Seminal vesicle invasion; LNI: Lymph node involvement; NOC: Non-organ confined disease; BCR: Biochemical recurrence; ROC: Receiver operation characteristic; AUC: Area under the curve; PCRC: Prostate cancer research consortium; BPH: Benign prostatic hyperplasia; GS: Gleason score; CI: Confidence interval; LR: Logistic regression; RF: Random forests; kNN: K nearest neighbours; PPV: Positive predictive value; NPV: Negative predictive value.

## Competing interests

The authors declare they have no completing interests.

## Authors’ contributions

RWW and TBM jointly contributed to conception and design. RWW developed the Prostate Cancer Research Consortium biobank and hence is responsible for the acquisition of data. SB carried out data preparation, data analysis and interpretation of data. YF assisted with the statistical analysis of the data. SB drafted and revised the manuscript, with TBM and RWW giving final approval. All authors read and approved the final manuscript.

## Pre-publication history

The pre-publication history for this paper can be accessed here:

http://www.biomedcentral.com/1472-6947/13/126/prepub
